# Supplementary material for: Urolithin A Alleviates Doxorubicin-Induced Senescence in Mesenchymal Stem Cells
Source: Int J Mol Sci. 2025 Oct 22;26(21):10257. doi: 10.3390/ijms262110257 (PMC12608000; doi:10.3390/ijms262110257)
Supplement: Supplementary file 1 [file ijms-26-10257-s001.zip › Supplement Figure S1.pptx]

## Slide 1
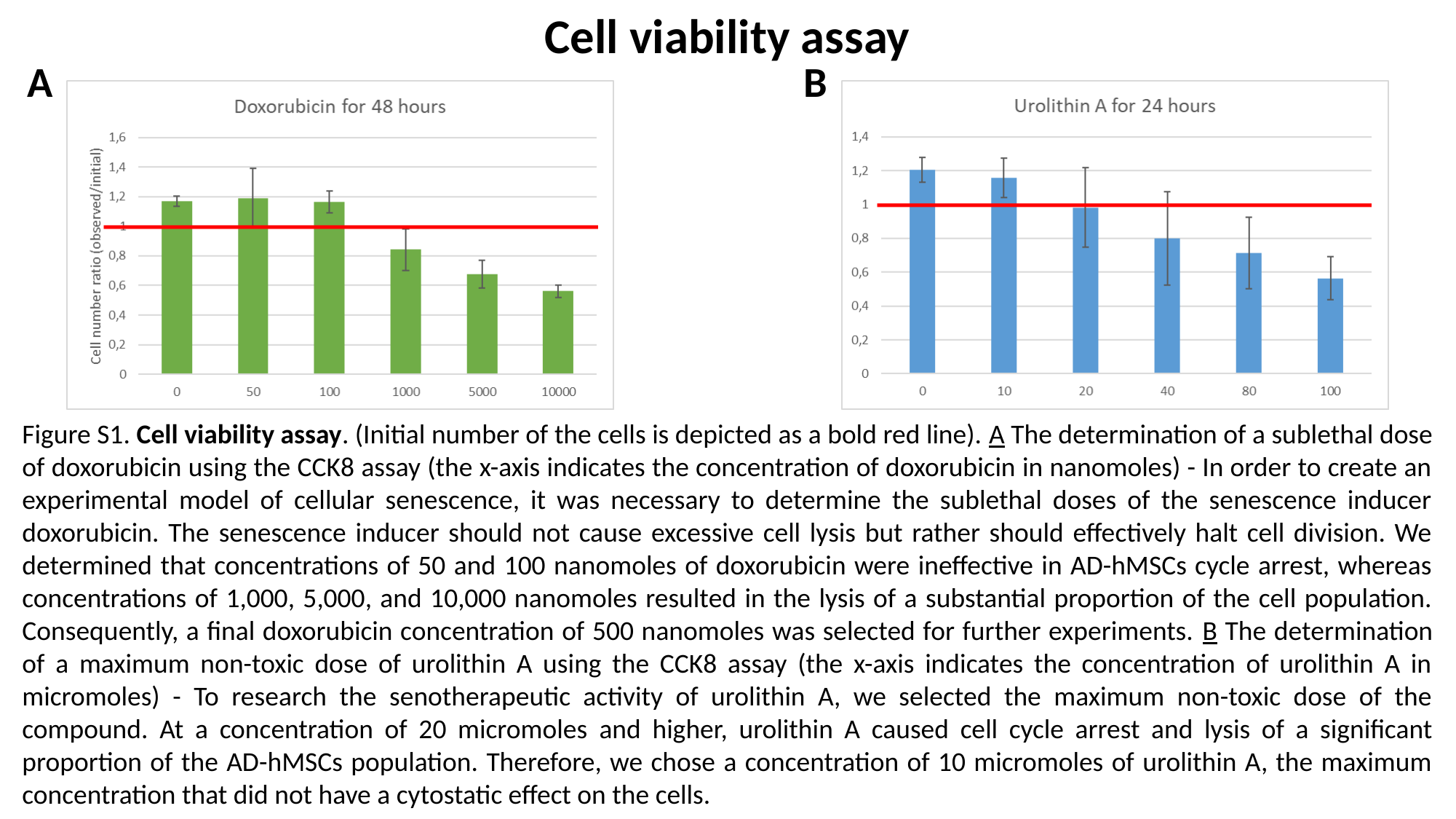

Cell viability assay
A
B
Figure S1. Cell viability assay. (Initial number of the cells is depicted as a bold red line). A The determination of a sublethal dose of doxorubicin using the CCK8 assay (the x-axis indicates the concentration of doxorubicin in nanomoles) - In order to create an experimental model of cellular senescence, it was necessary to determine the sublethal doses of the senescence inducer doxorubicin. The senescence inducer should not cause excessive cell lysis but rather should effectively halt cell division. We determined that concentrations of 50 and 100 nanomoles of doxorubicin were ineffective in AD-hMSCs cycle arrest, whereas concentrations of 1,000, 5,000, and 10,000 nanomoles resulted in the lysis of a substantial proportion of the cell population. Consequently, a final doxorubicin concentration of 500 nanomoles was selected for further experiments. B The determination of a maximum non-toxic dose of urolithin A using the CCK8 assay (the x-axis indicates the concentration of urolithin A in micromoles) - To research the senotherapeutic activity of urolithin A, we selected the maximum non-toxic dose of the compound. At a concentration of 20 micromoles and higher, urolithin A caused cell cycle arrest and lysis of a significant proportion of the AD-hMSCs population. Therefore, we chose a concentration of 10 micromoles of urolithin A, the maximum concentration that did not have a cytostatic effect on the cells.
