# Supplementary material for: Urolithin A Alleviates Doxorubicin-Induced Senescence in Mesenchymal Stem Cells
Source: Int J Mol Sci. 2025 Oct 22;26(21):10257. doi: 10.3390/ijms262110257 (PMC12608000; doi:10.3390/ijms262110257)
Supplement: Supplementary file 1 [file ijms-26-10257-s001.zip › Supplement Figure S2.pptx]

## Slide 1
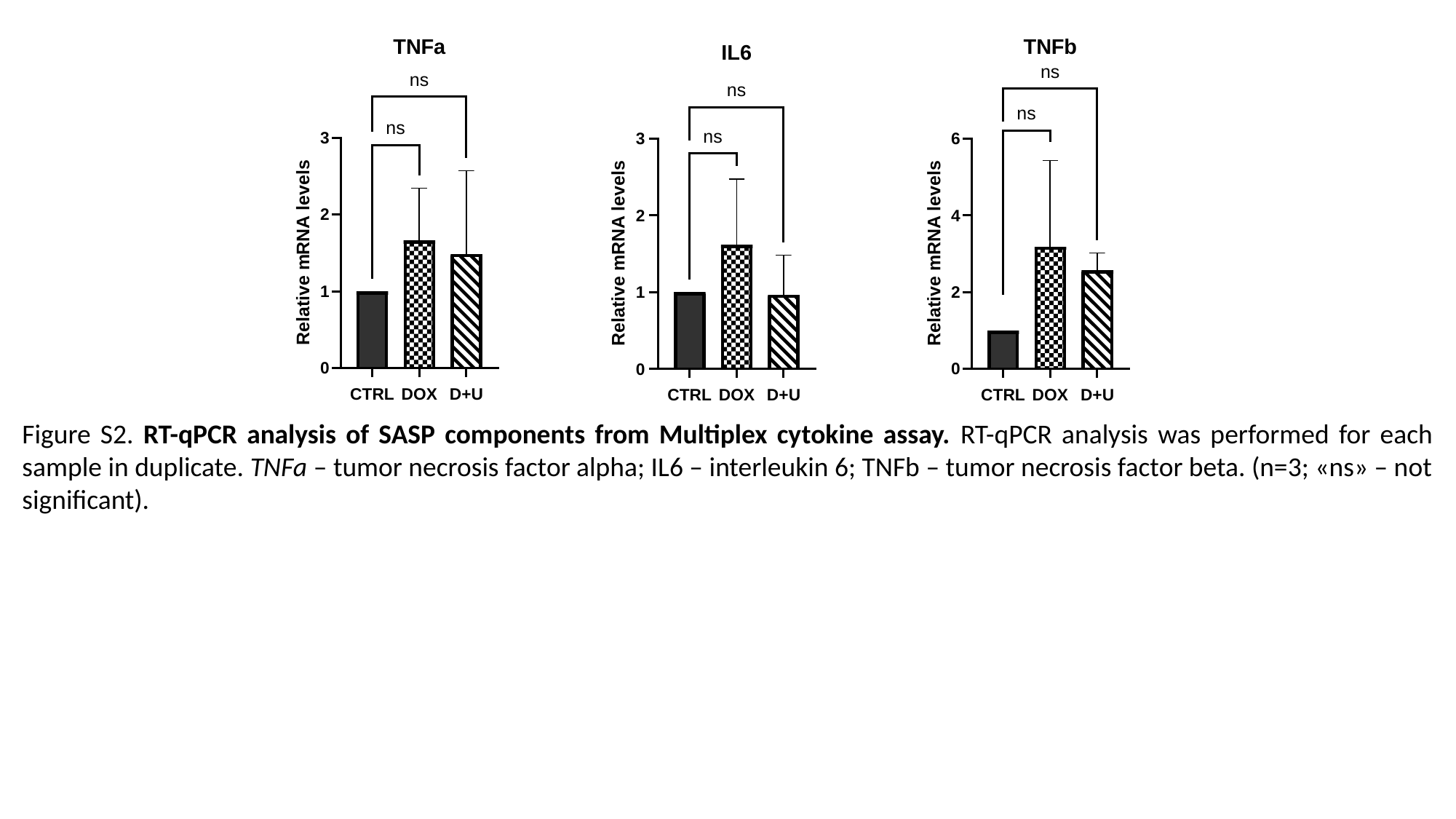

Figure S2. RT-qPCR analysis of SASP components from Multiplex cytokine assay. RT-qPCR analysis was performed for each sample in duplicate. TNFa – tumor necrosis factor alpha; IL6 – interleukin 6; TNFb – tumor necrosis factor beta. (n=3; «ns» – not significant).
